# Revisiting Neuroblastoma: Nrf2, NF-κB and Phox2B as a Promising Network in Neuroblastoma

**DOI:** 10.3390/cimb46040200

**Published:** 2024-04-06

**Authors:** Sara Peggion, Safiullah Najem, Jan Philipp Kolman, Konrad Reinshagen, Laia Pagerols Raluy

**Affiliations:** Department of Pediatric Surgery, University Medical Centre Hamburg-Eppendorf, Martinistrasse 52, 20246 Hamburg, Germany

**Keywords:** neuroblastoma, signaling network, genetic alterations, Nrf2, NF-κB, Phox2B

## Abstract

Neuroblastoma is the most common solid extracranial tumor during childhood; it displays extraordinary heterogeneous clinical courses, from spontaneous regression to poor outcome in high-risk patients due to aggressive growth, metastasizing, and treatment resistance. Therefore, the identification and detailed analysis of promising tumorigenic molecular mechanisms are inevitable. This review highlights the abnormal regulation of NF-κB, Nrf2, and Phox2B as well as their interactions among each other in neuroblastoma. NF-κB and Nrf2 play a key role in antioxidant responses, anti-inflammatory regulation and tumor chemoresistance. Recent studies revealed a regulation of NF-κB by means of the Nrf2/antioxidant response element (ARE) system. On the other hand, Phox2B contributes to the differentiation of immature sympathetic nervous system stem cells: this transcription factor regulates the expression of *RET*, thereby facilitating cell survival and proliferation. As observed in other tumors, we presume striking interactions between NF-κB, Nrf2, and Phox2B, which might constitute an important crosstalk triangle, whose decompensation may trigger a more aggressive phenotype. Consequently, these transcription factors could be a promising target for novel therapeutic approaches and hence, further investigation on their regulation in neuroblastoma shall be reinforced.

## 1. Introduction

Neuroblastoma derives from sympathetic nervous system stem cells and accounts for the most common solid extracranial tumor during childhood, accounting for 5.5% of all pediatric malignancies. Hence, 11 out of 1,000,000 children and adolescents between 0 and 17 years are diagnosed with this disease [1,2]. Different parameters have been used to classify patients with neuroblastoma into subgroups by their risk of death: age at diagnosis, metastatic spread, degree of tumor differentiation as well as histological features, mitosis-index, *MYCN* oncogene amplification, 1p/17q/11q status, DNA ploidy and LDH and ferritin levels [3,4]. 

For example, patients with high-risk disease (stage 4) are older than 18 months (median presentation age) and present metastases; their cancers show *MYCN* amplification, 1p deletion, 17q gain, 11q LOH, diploidy, as well as high LDH and ferritin levels—otherwise they are grouped to non-high-risk disease (stage 1–3) [5]. 

Children with stage 4 neuroblastoma, accounting for approximately 60%, have one of the worst prognoses in pediatric oncology [4]. Despite significant therapeutic improvements including high-dose chemotherapy, surgical resection, radiotherapy, immunotherapy, and myeloablative therapy with autologous stem cell transplantation, the 5-year overall survival rate remains below 50% in these patients [6,7,8,9]. 

Cancer development and progression are multifactorial processes in which several elements (both intrinsic and extra-tumoral) interact with one another. Accordingly, abnormal regulation of genetic factors, such as NF-κB, *MYCN*, *ALK*, and Nrf2*,* has been constantly and similarly observed in various cancer types [10]. Although neuroblastoma characterizes for a wide range of different chromosomal abnormalities and gene mutations [5], to date no common neuroblastoma-specific genomic alteration, LOH, or genetic translocation has been uniformly linked to all aggressive neuroblastoma phenotypes [11].

A *MYCN* amplification is displayed by approximately 18–38% of tumors and linked with the most aggressive disease course. Its alteration is associated with segmental loss of the distal short arm of chromosome 1 (1p). On the other hand, 11q deletion also correlates with poor prognosis, but shows a negative correlation with *MYCN* amplification [1,5].

Other factors, in turn, are altered in a tumor-specific manner and may consequently qualify as tumor biomarkers (e.g., GD2 and Phox2B for neuroblastoma) [12]. Despite the hitherto conducted efforts to characterize neuroblastoma, a proper interpretation of the genetic and epigenetic features of neuroblastoma is still missing.

The goal of this scope review is to shed light onto overlooked signaling pathways possibly involved in the carcinogenesis of neuroblastoma, with emphasis on Nrf2, NF-κB, and Phox2B. Additionally, we aim to uncover their interaction and contextualize it in neuroblastoma. We presume to portray and then partially fill the current knowledge gap regarding these functional networks. This review shall motivate for novel research approaches and deeper analyses ensuring better comprehension of these factors’ role in the context of neuroblastoma, with a focus on their possible striking role in the development of aggressive tumor traits. 

## 2. Nrf2 Signaling Pathway

### 2.1. Nrf2 Molecular and Functional Biology

The nuclear factor erythroid 2-related factor 2 (Nrf2) is a transcription factor, belonging to a family of basic leucine zipper (bZIP) proteins, a member of the cap‘n’collar (CNC) subfamily that in humans is encoded by the *NFE2L2* gene [13,14]. Nrf2 has seven functional domains (Nrf2-ECH homology, Neh1–7), which are either implicated in the regulation of Nrf2 stability or necessary for its transcriptional activity. It is primarily involved in response to oxidative stress, in fact regulating the expression of hundreds of cytoprotective genes which contain antioxidant response elements (ARE) in their promoters, such as *HO-1* [15,16].

**Figure 1:**
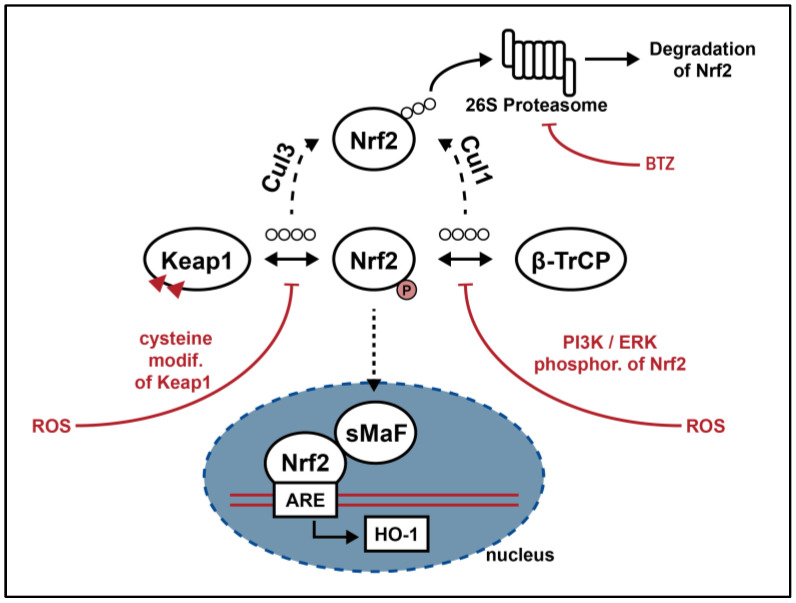
Regulatory axis of Nrf2: in basal conditions the interaction between Nrf2 and either Keap1 or β-TrcP leads to Nrf2 ubiquitination and proteasomal degradation. In cases of cell exposure to oxidative stress, cysteine modifications on Keap1 decrease its affinity to Nrf2, which translocate into the nucleus and, together with its cofactor sMaf, binds cytoprotective genes containing ARE in their promoters. PI3K/ERK-mediated phosphorylation of Nrf2 prevents its β-TrcP-driven ubiquitination, thus enabling its translocation into the nucleus too [16,18]. BTZ is a selective and reversible 26S proteasome inhibitor, able to improve Nrf2 activity by reducing its proteasomal degradation [16]. (↔︎ interaction; ⇢ translocation; ↳ transcription; ⊣ inhibition).

As depicted in Figure 1, different Nrf2-regulation pathways have been described. Traditionally the ETGE and DLG motifs of the N-terminal domain (Neh2) of Nrf2 bind two molecules of E3 ligase adaptor Kelch-like ECH-associated protein 1 (Keap1) under homeostatic conditions. This interaction activates the Cullin3 (Cul3) E3 ubiquitin ligase complex-mediated ubiquitination of Nrf2, which leads to its degradation by 26S proteasome [17,18]. In cases of oxidative damage, i.e., through cell exposure to exogenous chemicals, the cytoplasmatic concentration of ROS and electrophiles increases [19]. The resulting modification of cysteine residues of Keap1 reduces the affinity between Keap1 and Nrf2, leading to complex dissociation and the subsequent translocation of Nrf2 into the nucleus [18]. The phosphorylation of Nrf2 appears to be a further important event in the release of Nrf2 from Keap1 [20]. Another model displays a Keap1-independent regulation of Nrf2: under physiological conditions its Neh6 domain binds Beta-transducin repeats-containing protein (β-TrCP), enabling Nrf2 ubiquitination by Skp1-Cul1-Rbx1/Roc1 ubiquitin ligase complex and ultimately its proteasomal degradation [19]. The stability of this complex is jeopardized by phosphorylation of Nrf2, which is in turn initialized by elevated oxidative stress levels [21]. An additional regulation of Nrf2 is via phosphatidylinositol 3-kinase (PI3K) and Akt, which leads to Nrf2 activation by phosphorylation of its phosphatidylinositol at the D-3 position of the inositol ring [22]. 

Furthermore, the inhibition of proteasome activity can increase cytoplasmatic level of Nrf2 and indirectly its activity [16]. Recent studies have identified numerous proteins with motifs that are very similar to that of ETGE in Nrf2; these proteins might be able to compete with Nrf2 for Keap1 binding, thus stabilizing Nrf2 [14].

Once Nrf2 is in the nucleus, the bZip motif in the Neh1 domain of Nrf2 heterodimerizes with small musculoaponeurotic fibrosarcoma proteins (sMafs) and binds to ARE, initiating the transcription of downstream target genes. The resulting expressed proteins are involved in the response against stress and inflammation as well as in maintaining redox homeostasis. They can be classified into three major groups: phase I and phase II drug-metabolizing enzymes as well as phase III drug transporters [18]. 

Various studies outlined how the stimulus-induced activation of Nrf2 might be able to suppress carcinogenesis, at least in its early phase. Indeed, Nrf2-deficient mice have been shown to easily develop drug toxicity as well as a larger number of different tumors after exposure to exogenous chemicals/carcinogens compared to wild-type mice [18,23]. On the contrary, constitutive Nrf2 activation or its overactivation promotes carcinogenesis in plural ways: metabolic reprogramming, cell-cycle regulation [24], healthy mitochondrial maintenance [18,25], induction of angiogenesis [26], suppression of cell apoptosis [27], reduction of tumor-associated chronic inflammation, maintenance of the self-renewal potential of cancer stem cells, and contribution to chemo- and radio-resistance [16]. 

### 2.2. Nrf2 in Neuroblastoma

Although the oncogenic role of Nrf2 has been repeatedly highlighted, there is only fragmentary knowledge about its involvement in the carcinogenesis and development of neuroblastoma, mostly based on single experiments on neuroblastoma-derived cell lines. 

In neuroblastoma cells the activation of TRPM2, an ion channel permeable to Ca^2+^, K^+^, and Na^2+^, might induce the cAMP response element-binding protein(CREB)-mediated expression of Nrf2, thus promoting oxidative stress regulation and cell survival [28].

In the work of Kaufman et al. (2020), human IMR-32 neuroblastoma cells were exposed to high dopamine concentrations to induce oxidative stress. In the presence of sufficient zinc in the cell culture medium, this cell line responded by upregulating heme oxygenase 1 (HO-1), a downstream target of Nrf2 [29]. 

In highly chemo-resistant neuroblastoma cells HTLA-230, treatment with bortezomib (BTZ), the first selective and reversible 26S proteasome inhibitor, led to Nrf2 activation, thus reducing the expected improvement in cell chemosensitivity. BTZ usually increases the apoptotic rate of tumoral cells due to the enhanced response to chemotherapy. In the aforementioned neuroblastoma cells, the Nrf2-mediated upregulation of HO-1 and ultimately the increment of total glutathione (GSH) levels after BTZ exposure, prevented cell damage [16]. 

In the study of Wang et al. (2008), stable overexpression or induced upregulation of Nrf2 in SH-SY5Y neuroblastoma cells was reliably linked to enhanced resistance to cisplatin, etoposide and doxorubicin, which are utilized in the treatment regimen for high-risk neuroblastoma [30,31]. Chemicals which selectively inhibit the Nrf2 signal pathway might be used as adjuvants to chemotherapy in order to potentiate the efficacy of well-established treatment protocols [32,33]. So far, the only known selective Nrf2-inhibitor is brusatol, a quassinoid derived from the plant *Brucea sumatrana*, which was shown to sensitize cancer cells and xenografts to cheloid agents. Clinical application was limited by systemic toxicity in early phase clinical studies. Furthermore, its regulation on the Nrf2 pathway was never tested in neuroblastoma cells or samples [34,35]. 

De Miranda Ramos et al. (2016) used the cell line SH-SY5Y as a retinoic acid (RA)-induced differentiation model. RA acts as a morphogen, controlling tissue growth and differentiation through genomic and non-genomic actions. RA-treatment of SH-SY5Y cells induced Nrf2 activation via thiol-dependent signaling and a rapid non-genomic activation of PI3K/Akt and ERK1/2, leading to cell survival by augmented resistance to RA cytotoxicity [36]. The activation of the PI3K/AKT/Nrf2 pathway and increasing Nrf2-induced expression of HO-1 after ROS impairment in human neuroblastoma cells SH-SY5Y has also been reported in other studies [37,38]. Additionally, combined treatment with all-trans retinoic acid (ATRA) and 2-cyano-3,12-dioxooleana-1,9(11)-dien-28-oic acid (CDDO) on human IMR32 neuroblastoma cells and mouse neuroblasts Neuro2a induced differentiation and neuron outgrowth; this was accompanied by a decreased expression of *MYCN* in the case of IMR32 cells. CDDO belongs to the compound group of triterpenoids and their (semi)synthetic derivatives, which characterize for their anti-inflammatory and cytotoxic activity as well-known Nrf2 activators [39,40]. Another group member, soloxone methyl (SM), was used to treat the aforementioned murine Neuro2a neuroblastoma cells by Odarenko and colleagues, after cell stimulation with the carcinogenic leptine. Interestingly, their results showed that leptin-associated phosphorylation of ERK1/2 was suppressed by SM [41]. 

Alternatively, epigenetic therapeutic strategies may be considered. Although there is no direct proof of chromatin structure alterations in either *NFE2L2* or *KEAP-1* in neuroblastoma, there are a few studies supporting potential epigenetic modifications of both genes. Accordingly, Zhao et al. showed an increase of Nrf2 expression after treating murine neuroblastoma cells with sulforaphane (SFN), possibly mediated by decreased DNA demethylation of the *NFE2L2* promoter [42]. Moreover, other groups demonstrated a correlation between promoter methylation, histone acetylation status of *NFE2L2*/*KEAP-1* and the biological function of several tumor cells. Alike, the regulation of histone deacetyltransferases (HDACs) and DNA methyltransferases (DNMTs) were shown to modulate the expression and activity of Nrf2 and Keap-1 in prostate cancer, lung cancer and colorectal carcinoma [42,43,44]. In contrast to Zhao’s findings, in these tumor entities the hypermethylation on the *NFE2L2* promoter suppresses its transcription and leads to a subsequent inhibition of cancer progression. On the other hand, some cases of malignant glioma are characterized by an increase of *KEAP-1* promoter methylation which leads to an upregulation of *KEAP-1* transcription and a consequent higher degradation of Nrf2. In such cases, the therapeutic application of compounds such as Fumonisin B may induce cell death, as shown in hepatocellular carcinoma-derived cells. In this case, treatment of the HepG2 cell line with the aforementioned mycotoxin led to hypermethylation of the *KEAP-1*-promoter and to hypomethylation of *NFE2L2-*promoters. Consequently, decreased Keap-1 expression was observed, whereas Nrf2 was up-regulated [45].

## 3. *NF-kB* Signaling Pathway

### 3.1. NF-kB Molecular and Functional Biology

NF-κB (nuclear factor ‘kappa-light-chain-enhancer’ of activated B-cells) is a family of pleiotropic transcription factors expressed in all mammalian cells. This group comprises NF-κB1 (p50), NF-κB2 (p52), RelA (p65), RelB, and c-Rel. All of them share the Rel-homologue domain, but only the p65, c-Rel, and RelB proteins contain a transactivation domain (TAD), responsible for DNA binding. The functional biology of NF-κB differs based on which members of this superfamily form homo- or heterodimers [46,47].

Signal-induced activation of NF-κB occurs either through the canonical or the non-canonical pathway [47]. 

**Figure 2:**
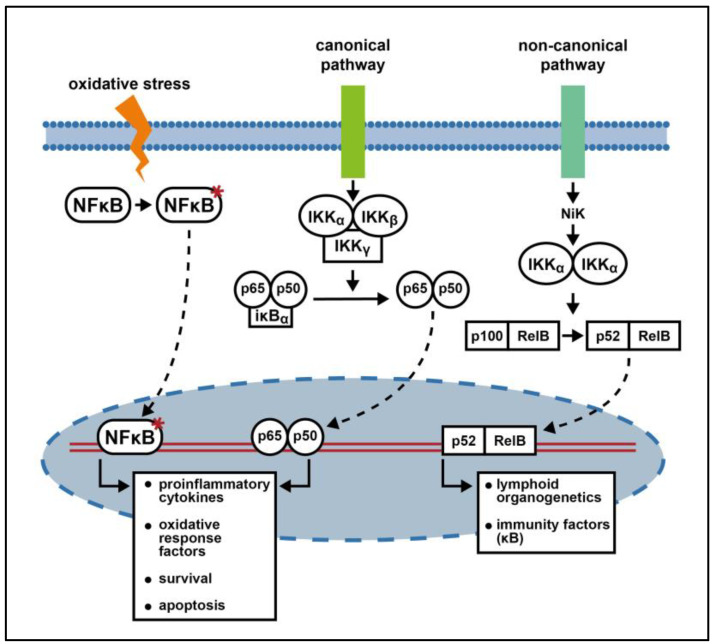
The regulatory axis of NF-κB: NF-κB activation can be mediated by three different pathways, depending on the activated membrane receptors. Each pathway leads to formation of a specific hetero- or homodimer, which in turn promotes the transcription of a distinct pattern of downstream targets, thus mediating pleomorphic effects [46,47]. (→ activation; ⇢ translocation; ↳ transcription; * phosphorylation).

In the canonical pathway (Figure 2), inactive dimers are anchored in the cytosol associated with one of the ankyrin-containing NF-κB inhibitors, the IκBs: IκBα, IκBβ, and IκBε [46]. Exposure to extracellular signals activates, for example, interleukin receptors, and TNF receptors type I as well as T- and B-cell receptors. Subsequent activation of the IκB kinase (IKK) complex, represented by IKK alpha, IKK beta, and IKK gamma/NEMO, leads to phosphorylation of IκBα, which undergoes ubiquitination and proteasomal degradation, freeing the NF-κB dimer p50/p65 [46,48]. Dimer translocation into the cell nucleus and adjunctive post-translational modifications are the final steps which permit DNA binding and the transcription of genes related to inflammation, cell survival, and proliferation (e.g., induction of IL-6, IL-8, Bcl-2, Fas, HO-1, BDNF and NGF) [28].

Interestingly, *IκBα* belongs to the downstream target genes of the canonical NF-κB activation cascade, thereby ensuring a negative control loop [47,49]. 

On the other hand, lymphotoxin B and B cell-activating factor (BAFF) are two of the few known factors able to induce the non-canonical pathway. In this pathway, NF-κB heterodimers contain p100, which is an inactive precursor of p52 [50]. Downstream, NIK (NF-κB-inducing kinase) is activated by phosphorylating the IKK-α dimer. Consequently, the NF-κB factor p100 is processed, turning into p52, allowing the heterodimer p52/RelB to translocate into the nucleus and activate the transcription of genes involved in immune cell development, such as clusters of differentiation 40 (CD40) and major histocompatibility complex type I (MHC I) [47,51,52]. 

Other alternative pathways responsible for NF-κB activation involve an oxidative stress response and rely on IKK-independent phosphorylation of IκBα [49]—(Figure 2).

As mentioned above, NF-κB has been identified as a pivotal mediator of inflammation and responsible for multiple innate and adaptative immune functions [53]. It also plays a crucial role in cell survival mechanisms: in tumors, the activation of NF-κB usually leads to impaired cell apoptosis and to the activation of genes regulating cell proliferation (cyclin D1/D2/D3). In most cancers, the activation of NF**-**κB is enhanced upon increased pathway stimulation, due to higher levels of TNFα and IL**-**1 in the tumor microenvironment [54]. 

Through analysis of in silico data from publicly available expression arrays, Medeiros and her team were able to depict increased NF-κB expression as a common denominator of pediatric brain tumors. Furthermore, NF-κB levels have been shown to be higher after chemo- or radiotherapeutic toxic aggression [55]. In fact, it has been reported that NF-κB also induces drug resistance in cancer cells, which is a major reason for therapy failure. Bentires-Aly and colleagues (2003) observed how NF-κB inhibition in HCT15 colon cancer cells led to an increased apoptosis rate after cell treatment with daunomycin, whose uptake was significantly increased [56].

### 3.2. NF-κB in Neuroblastoma

To date, neither chromosome rearrangements nor mutations are described for NF-κB in the context of neuroblastoma. In this tumor entity, NF-κB alterations are shown to be restricted to protein expression impairment, mostly induced. As for Nrf2, studies on NF-κB in neuroblastoma and its role as a possible therapeutic target are mostly based on in vitro assays. 

Nevertheless, its activity is linked to metastatic spread and regulation of the tumor microenvironment. TNFα-mediated activation of the NF-κB signaling pathway leads to upregulation of CX chemokine receptor-4 (CXCR4) expression in neuroblastoma samples compared to ganglioneuroma tissue specimens. CXCR4 fosters tumor metastasis, since it enables the homing in of cancer cells to organs expressing its ligand CXCL12, such as liver and bone marrow. Analogous results were observed in SH-SY5Y cells treated with TNFα, which displayed overexpression of both NF-κB and CXCR4. Contrarily, inhibition of NF-κB activity led to a reduced cell migration capacity [36].

As already mentioned, a further key role assigned to NF-κB is the regulation of apoptosis. Programmed cell death is a very complex and multifactorial process which is usually compromised in cancer; the role of NF-κB in apoptosis is equally complex. Both apoptosis suppression and induction have been reported for this transcription factor. Bian and colleagues showed that doxorubicin-induced apoptotic cell death in N-type neuroblastoma cells was mediated by NF-κB activation [56]. 

In SH-SY5Y cells, for instance, treatment with acetaminophen (AAP) resulted in the generation of ROS and the subsequent translocation of the NF-κB subunit p65 into the nucleus. This, in turn, led to the production of IL-1β with concomitant enhanced apoptosis [56,57,58]. Karacay et al. showed an NF-κB-mediated anti-apoptotic effect in several neuroblastoma cell lines. Transfection of these cancer cells with the *TRAIL* (TNF-related apoptosis-inducing ligand) gene led to apoptosis. However, the cell death rate was increased in those cells in which inhibition of the NF-κB signaling pathway occurred simultaneously. Strikingly, TRAIL is described as an inducer of NF-κB and thus it may be assumed that this ligand possibly act as a double-edged sword [59]. 

The ability of NF-κB to regulate apoptosis, together with the transcriptional regulation of immune-related genes, lend neuroblastoma cells the advantage of evading the immune system and developing treatment resistance. Transcriptional inhibition of NF-κB downregulates MHC Class I expression in human neuroblastoma cells [57,60]. To the downstream genes of NF-κB also belongs *MYCN:* N-myc is suppressed after treating neuroblastoma cells with NF-κB inhibitor withanolide [61]. 

Another known NF-κB inhibitor is dimethylamino parthenolide (DMAPT), which induces the expression of two methyltransferases, i.e., SETD2 and NSD1; the latter is epigenetically silenced in neuroblastoma and functions therefore as a tumor suppressor [62].

## 4. Phox2B Signaling Pathway

### 4.1. Phox2B Molecular and Functional Biology

Paired-like homeobox 2b gene (*PHOX2B*) is located on chromosome 4p12; it consists of 3 exons and encodes for a protein of 314 amino acids, i.e., a transcription factor which is a fundamental regulator of the initial differentiation and development of neural crest cells. Phox2B is expressed in sympathetic, parasympathetic and enteric ganglia of the developing peripheral autonomic nervous system. Up to date, very little is known about the regulation of Phox2B. Bone morphogenetic protein (BMP) signaling released by the dorsal aorta induces the neural crest cells to express high levels of Phox2B, and an interplay between microRNA(miR)-204, *MYCN* and *PHOX2B* has been described [63]. Whereas *MYCN* positively controls *PHOX2B* transcriptional expression, miR-204 targets *PHOX2B* leading to its downregulation [64,65]. *PHOX2B*, on the other hand, regulates the Delta–Notch pathway leading cells towards differentiation. Upstream, the helix–loop–helix transcription factor MASH1, a pro-neural factor promoting neuronal differentiation, and several genes such as *PHOX2A*, *ALK* and *RET* show activity in enteric neuron precursors [63]. At the same time, Phox2A acts as a transcriptional regulator of Phox2B [66]. Interestingly, the NF-κB pathway is involved in *PHOX2B* transcriptional regulation: the inhibition of NF-κB causes the accumulation of Nestin, Sox2, and glial fibrillary acidic protein with the consequent maintenance of neural stem cells in an undifferentiated state [67]. 

### 4.2. Phox2B in Neuroblastoma

Mutations in *PHOX2B* lead to neurodevelopmental disorders and have been linked to several congenital human diseases, including congenital central hypoventilation syndrome and Hirschsprung’s disease [68,69,70,71]. Additionally, in the last decade *PHOX2B* has moved to the main genetic landscape of neuroblastoma [69,72], becoming a reliable diagnostic marker in tissue and cytology specimens [12,73], and a specific and sensitive biomarker for minimal residual disease in neuroblastoma [74]. Indeed, a heterogenic pool of mutations in the PHOX2B gene have been observed in familial and sporadic forms of neuroblastoma, but in most neuroblastoma cells *PHOX2B* is highly expressed rather than downregulated [75]. Neuroblastoma exclusive *PHOX2B* mutations are missense mutations occurring in exon 1, frameshift mutations arising in exon 1 and 3 and unbalanced gene expressions [76]. Syndromic neuroblastoma is caused by mutations in the homeodomain and in exon 3. Some missense mutations lead to a decrease of Phox2B-mediated genes transcription [77]. This is also the case of frameshift mutations which either lead to elongated proteins or truncated Phox2B proteins [78]. *PHOX2B* mutations do impair inhibition of MSX1 (Msh Homeobox 1), thus resulting in upregulation of the Delta–Notch pathway, *NOTCH3* overexpression and activation, and aberrant transcriptional regulation of the anaplastic lymphoma kinase (*ALK*) gene by directly binding its promoter [72,79,80]. Among the many transcription factors involved at different neuroblastoma differentiation stages, *PHOX2B* and *MYCN* with their transcriptional targets *ALK* and *LIN28B*, and the tumor suppressor miRNAs let-7, miR-34, and miR-204 are mainly involved [65,81]. Ke et al. presented that *MYCN* expression promotes the proliferation of *PHOX2B*-positive neuronal progenitors, thus forming hyperplastic lesions in sympathetic ganglia, and surmised this for the initiation and progression of neuroblastoma development [82]. Reiff et al. provided the first demonstration that *PHOX2B* mutations give rise to gain-of-function protein variants that stimulate the proliferation of potential tumor founder cells of the sympathoadrenergic lineage. Thus, they speculated that tumorigenesis may rely on the acquisition of novel, proliferation-stimulating properties, and that impaired differentiation [83]. However, *PHOX2B* also has tumor suppressor properties in the final metastatic phase: knockdown of *PHOX2B* in human neuroblastoma micrometastasis increased the metastatic potential of the cells, which were orthotopically inoculated in nude mice [84].

Based on the fact that *PHOX2B* transcription is regulated by the tumor microenvironment [84,85], Di Zanni et al. considered downregulation of *PHOX2B* expression by treating neuroblastoma cells with pharmacological compounds. Incubation of cell lines with mycophenolate mofetil and chloroquine, which negatively act on *PHOX2B* promoters, led to decreased cell proliferation and increased apoptosis and cell differentiation [67]. 

Similarly, de Pontual et al. showed that the differential expression of *PHOX2B* in several human neuroblastoma cell lines and tumor samples partially relied on the methylation-mediated silencing of its promoter. Among all 13 studied cell lines, SK-N-BE, GIMEN, and SK-N-SH expressed no Phox2B, and only SK-N-BE and GIMEN were able to re-express it after treatment with 5’-aza, a methyltransferase inhibitor [86]. 

## 5. Potential Nrf2—NF-κB—Phox2B Crosstalk in Neuroblastoma

**Figure 3:**
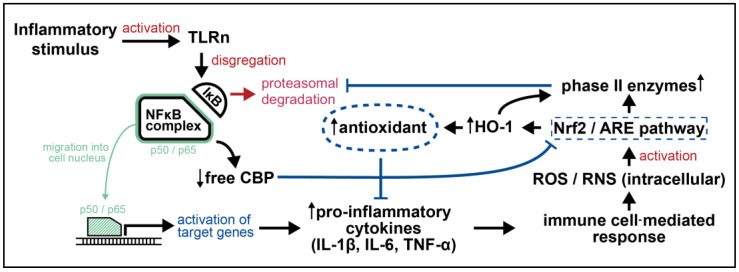
**Mutual interaction between Nrf2 and NF-κB pathways in a proinflammatory state:** CBP is a crucial intersection between the two axes. CBP is able to acetylate histone and promote chromatin remodeling, which is mandatory for NF-κB to start its translational activity. At the same time CBP-mediated acetylation of Nrf2 is necessary to allow DNA-binding. Nrf2 and NF-κB compete for the same co-activator. The NF-κB member p65 shows higher affinity for CBP than Nrf2: in the case of overexpression and/or overactivation of p65, the level of free CBP decreases, thereby downregulating Nrf2 transcriptional activity [19,87,88]. (→ activation; ↳ transcription; ⊣ inhibition; ↑ up-regulation; ↓ down-regulation).

Inflammation, as a response against tissue homeostasis impairment, is involved in a plethora of pathologic processes [13]. After signal recognition, Toll-like receptors (TLRs) mediate the activation of specific immune cascades leading to NF-κB dissociation from IκB. Subsequently, NF-κB is able to translocate into the nucleus, where it acts as a transcription factor, eventually upregulating the secretion of pro-inflammatory cytokines (e.g., IL-1β, IL-6 and, TNF-α) [69]. Consequent recruitment of immune cells at an injury site results in increased ROS and reactive nitrogen species (RNS). Since Nrf2-signaling plays a key role in redox homeostasis by reducing intracellular ROS levels, it might counteract NF-κB effects under determined conditions. A further regulatory mechanism is represented by Nrf2-mediated overexpression of phase II enzymes which block proteasomal degradation of IκB-α, necessary for NF-κB translocation into the nucleus [70]. An additional attractive crosstalk point between the two cascades has been found in CREB binding protein (CBP), which is a transcription co-activator of NF-κB, able to acetylate histones for chromatin remodeling as well as non-histone proteins [71]; in the case of Nrf2, this is a mandatory step for Neh1-domain-mediated DNA-binding. Similar to NF-κB, CBP acts as a transcriptional co-activator of Nrf2, binding its Neh4 and Neh5 domains, nevertheless with a lower affinity than to p65. Thus, both transcription factors compete for CBP accessibility: when p65 is overexpressed the amount of free CBP for Nrf2 is reduced and, hence, Nrf2-mediated gene transcription decreases, as depicted in Figure 3 [13].

To the best of our knowledge this multifaceted network has not been systematically investigated in neuroblastoma yet. A limited number of studies utilized SH-SY5Y human neuroblastoma cells to test the neuroprotective effect of multiple compounds, such as glucomoringin isothiocyanate (GMG-ITC), Klotho, Tavarua Deoxyriboside A, and Jasplakinolide [89,90,91]. The common denominator among these substances is their ability to inhibit the NF-κB signaling pathway while activating the Nrf2/ARE system. This crosstalk might be even more byzantine, considering the possible linkage between the aforementioned cascades and the paired-like homeobox 2B (*PHOX2B*) pathway via anaplastic lymphoma kinase (ALK). 

As already underlined, various research works on SH-SY5Y cells suggested a rapid non-genomic activation of the Nrf2 axis mediated by PI3K/Akt and ERK1/2 [36,37,38]. Activation of ALK stimulates downstream signaling through both the PI3K/Akt and the MAPK/ERK cascade. Therefore, it may be presumed that an overactivation or overexpression of ALK indirectly leads to a reinforced activity of Nrf2 [92]. ALK-activating mutations have been detected in most familial and in 10% of sporadic neuroblastoma cases [93]. Concordantly, Heukamp et al. were able to induce neuroblastoma in transgenic mice through targeted expression of the most aggressive variant, ALKF1174. Chromosomal aberrations in this model, e.g., 17q gain and *MYCN* amplification, resembled those in human high-risk neuroblastoma [94]. Additionally, in the work of Lopez-Delisle et al. neuroblastoma patients with mutation-driven ALK activation showed poorer outcome than patients with non-mutated ALK [95]. An additional regulating element of *ALK* is Phox2B, since *ALK* is the major known target gene of Phox2B transcriptional activity [67]. 

In patients with neuroblastoma, different heterozygous missense and nonsense germline mutations of *PHOX2B* have been detected [69,76]. Its pathogenic role is traditionally associated with gene overexpression, which can in turn increase ALK expression and activity [67]. ALK upregulation might also be associated to a co-amplification with *MYCN*, either due to their proximity on 2p23, or as a direct transcriptional target of N-Myc [93]. The latter represents one of the most important hallmarks of high-risk neuroblastoma. The overmentioned PI3K was shown to stabilize N-Myc in mouse models for pediatric neural cancers [96]; PI3K overactivation has been reported in both neuroblastoma samples and cell lines, although its mutations are quite rare. Guo et al. have been able to correlate high expression of ALK subunit p110α with worse treatment outcomes of neuroblastoma patients by analyzing gene expression data from primary neuroblastoma samples [97]. PI3K is usually recruited to the cell membrane by transmembrane receptor tyrosine kinases (RTKs) after binding with specific growth factors. A further and crucial step is the activation of Akt. Opel et al. first provided evidence that Akt activation correlates with poor outcome by analyzing 116 primary neuroblastoma samples [98]. More recent studies on multiple neuroblastoma cell lines demonstrated how inhibitors of Akt (e.g., Hu7691 [99]) or PI3K (e.g., BI-D1870 [100]) induced suppression of cell growth. Moreover, it shall not be forgotten that Akt is able to regulate activation of other transcription factors, e.g., NF-κB [101]. It is noteworthy that NF-κB signaling is required for neural stem cells’ early differentiation, as it is involved in *PHOX2B* transcriptional regulation. In fact, inhibition of NF-κB in transgenic mice maintains neural stem cells in an undifferentiated state [102]. This transcription factor also controls the transcription of *RET*, another RTK such as PI3K. RET is indispensable for physiologic development of sympathetic neurons [103] and its inactivating mutations and polymorphisms are a major cause of Hirschsprung’s disease, defined by colonic aganglionosis [104]. In addition, RET interacts with ALK and TRK, likewise members of the RTK family [103]. Recently it has been more precisely defined that RET hyperactivation is driven by ALK in neuroblastoma [95]. In SH-SY5Y neuroblastoma cells this regulation seems to be mediated through the PI3K/AKT pathway, which in turn can be activated by RET. Indeed, the RET-induced activation of RAS/ERK and PI3K/Akt would lead to hyperfunction of both Nrf2 and NF-κB pathways, which seem to primarily inhibit each other, shedding light on the complexity of the crosstalk between Phox2B and both Nrf2 and NF-κB cascades [105]. 

According to the proposed interactions, it may be possible to presume a shared therapeutic pattern in neuroblastoma in which a few compounds regulate at least two of the hereby described transcription factors. Some groups have shown the effects of phytocompounds, such as sulforaphane and luteolin, on the methylation status of *NFE2L2*/*KEAP-1* promoters, thereby influencing cell survival and tumor progression in different cancer entities [43,106]. Luteolin regulates not only the Nrf2 axis via epigenetic modulation, but also inhibits NF-κB, thereby blocking the degradation of IκBα [43]. The use of 5´-aza may also be considered in neuroblastoma treatment due to its regulatory effect on the activity of both *KEAP-1* and *PHOX2B* promoters [86]. 

In order to reinforce the importance of analyzing the NF-κB—Nrf2—Phox2B network we used the well-known core data source STRING, which shows active and predicted protein–protein interactions based on a given query. These interactions include both physical and functional associations and rely on sources like laboratory experiments data, automated text mining and previous knowledge in datasets [107]. 

**Figure 4:**
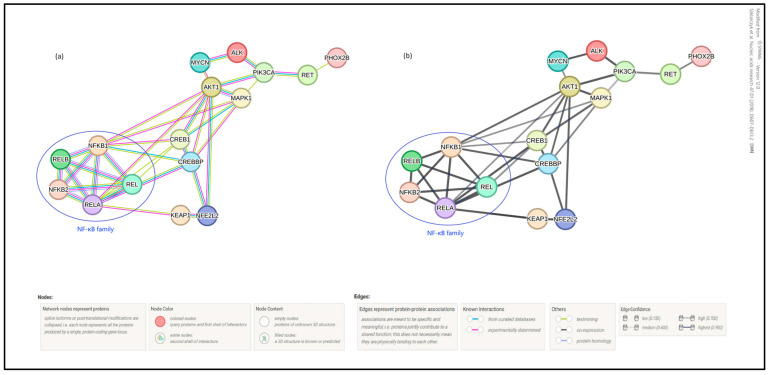
**STRING-derived protein–protein interaction network:** inserting in the STRING research tool the main proteins and genes highlighted in the review and filtering for interactions with a high minimum-required interaction score (>0.750—high confidence), the STRING algorithm system outputs a network in which all elements are linked. In (**a**) different levels of interaction are shown; in (**b**) the line thickness and grey color intensity indicate the strength of data support [108].

Introducing Phox2B, NF-κB (family members), Nrf2 (encoded as *NFE2L2*), Keap1, CREB/CREB binding protein (CBP), Akt/ERK, PI3K, ALK and *MYCN* in the STRING search tool and filtering for interactions with a high minimum-required interaction score (>0.750—high confidence), the STRING algorithm system output the scheme depicted in Figure 4. STRING also showed differentiated interaction confidence scores, based on each of the aforementioned sources. In the case of RelA and Nrf2, for instance, a direct interaction at the experimental level accounted for a score of 0.322. As described above, both transcription factors share —at least—the two binding proteins Keap1 and CBP (with respective purely experimental confidence scores of 0.370/0.991 for RelA and of 0.999/0.625 for Nrf2). Interestingly, NF-κB (RelA/p65) and Nrf2 are involved in both metabolic and in stress pathways. 

Subsequently, CREB/CBP, Nrf2 and RelA converge in AKT1 which, in turn, affiliates with *MYCN* with a combined score of 0.798. At this point, *MYCN*, ALK, RET and Phox2B built a complex network involved in different types of cancer, mostly involving the nervous system, also in regard to NF-κB (RelA/p65). 

By screening for common GO pathways (Gene Ontology—see Supplementary Material), NF-κB (RelA/p65), Nrf2, and Phox2B join in the regulation of metabolic processes. Furthermore, Nrf2 and Phox2B share a role in the regulation of neuron projection development, whereas RelA/p65 and Nrf2 are functionally close in the regulation of the immunological and inflammatory response, especially to reactive oxygen species (Figure 4). 

## 6. Conclusions

This review outlines the molecular and functional biology of the three transcription factors Nrf2, NF-κB and Phox2B, insinuating the role of their interaction in the development, progression and aggressive behaviour of neuroblastoma. 

Unlike Phox2B, which is well described in the context of neuroblastoma, it is not possible to clearly infer the potential actions of Nrf2 and NF-κB in the molecular machinery of this cancer type. Based on recent studies and extrapolating the regulation patterns from other tumor entities, hereby we have intended to depict a structural and functional model in which Nrf2, NF-κB and Phox2B may compose a transcription factor network. The regulation of it may be finely orchestrated by a wide range of stimuli interacting with several receptors. 

Considering neuroblastoma as the result of a dysregulated network rather than the sum of multiple genetic alterations, we encourage the screening and analysis of neuroblastoma in a more comprehensive way, allowing better patient stratification and therefore optimized identification of novel therapeutic targets. 

Starting from the analysis we performed with STRING, the development of a highly stringent computational model for the hereby presented networks might be a decisive next step, as shown by Lombardo et al. for PD-L1 expression in neuroblastoma [108].

In summary, a structured description of understudied signaling pathways in neuroblastoma might exert a better understanding of tumor progression and drug resistance in aggressive neuroblastoma phenotypes and, thus, facilitate the individuation of novel therapeutic targets.

## Supplementary Materials

The following supporting information can be downloaded at: https://www.mdpi.com/article/10.3390/cimb46040200/s1, File S1: Gene ontology dataset of the STRING-derived protein–protein interaction network displayed in Figure 4.

## References

[1] Matthay K.K., Maris J.M., Schleiermacher G., Nakagawara A., Mackall C.L., Diller L., Weiss W.A. (2016). Neuroblastoma. Nat. Rev. Dis. Primers.

[2] Erdmann F., Kaatsch P., Grabow D., Spix C. (2020). ‘German Childhood Cancer Registry—Annual Report 2019 (1980–2018).

[3] Shimada H., Chatten J., Newton W.A., Sachs N., Hamoudi A.B., Chiba T., Marsden H.B., Misugi K. (1984). Histopathologic Prognostic Factors in Neuroblastic Tumors: Definition of Subtypes of Ganglioneuroblastoma and an Age-Linked Classification of Neuroblastomas. JNCI J. Natl. Cancer Inst..

[4] Zafar A., Wang W., Liu G., Wang X., Xian W., McKeon F., Foster J., Zhou J., Zhang R. (2020). Molecular targeting therapies for neuroblastoma: Progress and challenges. Med. Res. Rev..

[5] Aygun N. (2018). Biological and Genetic Features of Neuroblastoma and Their Clinical Importance. Curr. Pediatr. Rev..

[6] Berthold F., Spix C., Kaatsch P., Lampert F. (2017). Incidence, Survival, and Treatment of Localized and Metastatic Neuroblastoma in Germany 1979–2015. Pediatr. Drugs.

[7] Park J.R., Bagatell R., London W.B., Maris J.M., Cohn S.L., Mattay K.M., Hogarty M., on behalf of the COG Neuroblastoma Committee (2013). Children’s Oncology Group’s 2013 blueprint for research: Neuroblastoma. Pediatr. Blood Cancer.

[8] Pinto N.R., Applebaum M.A., Volchenboum S.L., Matthay K.K., London W.B., Ambros P.F., Nakagawara A., Berthold F., Schleiermacher G., Park J.R. (2015). Advances in Risk Classification and Treatment Strategies for Neuroblastoma. J. Clin. Oncol..

[9] Berlanga P., Cañete A., Castel V. (2016). Advances in emerging drugs for the treatment of neuroblastoma. Expert Opin. Emerg. Drugs.

[10] Megison M.L., Gillory L.A., Beierle E.A. (2013). Cell Survival Signaling in Neuroblastoma. Anti-Cancer Agents Med. Chem..

[11] Louis C.U., Shohet J.M. (2015). Neuroblastoma: Molecular pathogenesis and therapy. Annu. Rev. Med..

[12] Ma Y., Feng J., Zhao J., Ding D., Tian F., Chen L., Zheng J., Xiao X. (2021). PHOX2B as a Reliable Marker for Neuroblastoma in Tissue and Cytology Specimens. J. Neuropathol. Exp. Neurol..

[13] Kobayashi M., Yamamoto M. (2005). Molecular Mechanisms Activating the Nrf2-Keap1 Pathway of Antioxidant Gene Regulation. Antioxidants Redox Signal..

[14] Krajka-Kuźniak V., Paluszczak J., Baer-Dubowska W. (2017). The Nrf2-ARE signaling pathway: An update on its regulation and possible role in cancer prevention and treatment. Pharmacol. Rep..

[15] Ahmed S.M.U., Luo L., Namani A., Wang X.J., Tang X. (2017). Nrf2 signaling pathway: Pivotal roles in inflammation. Biochim. Biophys. Acta Mol. Basis Dis..

[16] Furfaro A.L., Piras S., Domenicotti C., Fenoglio D., De Luigi A., Salmona M., Moretta L., Marinari U.M., Pronzato M.A., Traverso N. (2016). Role of Nrf2, HO-1 and GSH in Neuroblastoma Cell Resistance to Bortezomib. PLoS ONE.

[17] Sekhar K.R., Rachakonda G., Freeman M.L. (2010). Cysteine-based regulation of the CUL3 adaptor protein Keap1. Toxicol. Appl. Pharmacol..

[18] Wu S., Lu H., Bai Y. (2019). Nrf2 in cancers: A double-edged sword. Cancer Med..

[19] Saha S., Buttari B., Panieri E., Profumo E., Saso L. (2020). An Overview of Nrf2 Signaling Pathway and Its Role in Inflammation. Molecules.

[20] Huang Y., Li W., Su Z.-Y., Kong A.-N.T. (2015). The complexity of the Nrf2 pathway: Beyond the antioxidant response. J. Nutr. Biochem..

[21] Rada P., Rojo A.I., Chowdhry S., McMahon M., Hayes J.D., Cuadrado A. (2011). SCF/β-TrCP Promotes Glycogen Synthase Kinase 3-Dependent Degradation of the Nrf2 Transcription Factor in a Keap1-Independent Manner. Mol. Cell. Biol..

[22] Wang L., Chen Y., Sternberg P., Cai J. (2008). Essential Roles of the PI3 Kinase/Akt Pathway in Regulating Nrf2-Dependent Anti-oxidant Functions in the RPE. Invest. Ophthalmol. Vis. Sci..

[23] Ramos-Gomez M., Kwak M.-K., Dolan P.M., Itoh K., Yamamoto M., Talalay P., Kensler T.W. (2001). Sensitivity to carcinogenesis is increased and chemoprotective efficacy of enzyme inducers is lost in *nrf2* transcription factor-deficient mice. Proc. Natl. Acad. Sci. USA.

[24] Reddy N.M., Kleeberger S.R., Bream J.H., Fallon P.G., Kensler T.W., Yamamoto M., Reddy S.P. (2008). Genetic disruption of the Nrf2 compromises cell-cycle progression by impairing GSH-induced redox signaling. Oncogene.

[25] Holmström K.M., Baird L., Zhang Y., Hargreaves I., Chalasani A., Land J.M., Stanyer L., Yamamoto M., Dinkova-Kostova A.T., Abramov A.Y. (2013). Nrf2 Impacts Cellular Bioenergetics by Controlling Substrate Availability for Mitochondrial Respiration. Biol. Open.

[26] Shibuya A., Onda K., Kawahara H., Uchiyama Y., Nakayama H., Omi T., Nagaoka M., Matsui H., Hirano T. (2010). Sofalcone, a gastric mucosa protective agent, increases vascular endothelial growth factor via the Nrf2-heme-oxygenase-1 dependent pathway in gastric epithelial cells. Biochem. Biophys. Res. Commun..

[27] Niture S.K., Jaiswal A.K. (2012). Nrf2 Protein Up-regulates Antiapoptotic Protein Bcl-2 and Prevents Cellular Apoptosis. J. Biol. Chem..

[28] Miller B.A. (2019). TRPM2 in Cancer. Cell Calcium.

[29] Kaufman Z., Salvador G., Liu X., Oteiza P. (2020). Zinc and the modulation of Nrf2 in human neuroblastoma cells. Free. Radic. Biol. Med..

[30] Kim J.-Y., Surh Y.-J. (2009). The Role of Nrf2 in Cellular Innate Immune Response to Inflammatory Injury. Toxicol. Res..

[31] High-Risk Neuroblastoma Standard Clinical Practice Recommendations. https://siope.eu/media/documents/escp-high-risk-neuroblastoma-standard-clinical-practice-recommendations.pdf.

[32] Taguchi K., Motohashi H., Yamamoto M. (2011). Molecular mechanisms of the Keap1-Nrf2 pathway in stress response and cancer evolution. Genes Cells.

[33] Wang X.-J., Sun Z., Villeneuve N.F., Zhang S., Zhao F., Li Y., Chen W., Yi X., Zheng W., Wondrak G.T. (2008). Nrf2 enhances resistance of cancer cells to chemotherapeutic drugs, the dark side of Nrf2. Carcinogenesis.

[34] Cai S.J., Liu Y., Han S., Yang C. (2019). Brusatol, an NRF2 inhibitor for future cancer therapeutic. Cell Biosci..

[35] Ren D., Villeneuve N.F., Jiang T., Wu T., Lau A., Toppin H.A., Zhang D.D. (2011). Brusatol enhances the efficacy of chemotherapy by inhibiting the Nrf2-mediated defense mechanism. Proc. Natl. Acad. Sci. USA.

[36] Ramos V.d.M., Gasparotto J., Figueiró F., Dias A.d.F., Rostirolla D.C., Somensi N., da Rosa H.T., Grun L.K., Barbé-Tuana F.M., Gelain D.P. (2019). Retinoic acid downregulates thiol antioxidant defences and homologous recombination while promotes A549 cells sensitization to cisplatin. Cell. Signal..

[37] He D., Fu S., Zhou A., Su Y., Gao X., Zhang Y., Huang B., Du J., Liu D. (2021). Camptothecin Regulates Microglia Polarization and Exerts Neuroprotective Effects via Activating AKT/Nrf2/HO-1 and Inhibiting NF-κB Pathways In Vivo and In Vitro. Front. Immunol..

[38] Yang R., Ma S., Zhuo R., Xu L., Jia S., Yang P., Yao Y., Cao H., Ma L., Pan J. (2022). Suppression of endoplasmic reticulum stress-dependent autophagy enhances cynaropicrin-induced apoptosis via attenuation of the P62/Keap1/Nrf2 pathways in neuroblastoma. Front. Pharmacol..

[39] Chaudhari N., Ravanan P. (2018). Bardoxolone methyl induces neuritogenesis in Neuro2a cells. Pharmacol. Rep..

[40] Chaudhari N., Talwar P., D’Hellencourt C.L., Ravanan P. (2017). CDDO and ATRA Instigate Differentiation of IMR32 Human Neuroblastoma Cells. Front. Mol. Neurosci..

[41] Odarenko K.V., Salomatina O.V., Chernikov I.V., Salakhutdinov N.F., Zenkova M.A., Markov A.V. (2023). Soloxolone Methyl Reduces the Stimulatory Effect of Leptin on the Aggressive Phenotype of Murine Neuro2a Neuroblastoma Cells via the MAPK/ERK1/2 Pathway. Pharmaceuticals.

[42] Zhao F., Zhang J., Chang N. (2018). Epigenetic modification of Nrf2 by sulforaphane increases the antioxidative and anti-inflammatory capacity in a cellular model of Alzheimer’s disease. Eur. J. Pharmacol..

[43] Kang K.A., Piao M.J., Hyun Y.J., Zhen A.X., Cho S.J., Ahn M.J., Yi J.M., Hyun J.W. (2019). Luteolin promotes apoptotic cell death via upregulation of Nrf2 expression by DNA demethylase and the interaction of Nrf2 with p53 in human colon cancer cells. Exp. Mol. Med..

[44] Wang C., Shu L., Zhang C., Li W., Wu R., Guo Y., Yang Y., Kong A. (2018). Histone Methyltransferase Setd7 Regulates Nrf2 Signaling Pathway by Phenethyl Isothiocyanate and Ursolic Acid in Human Prostate Cancer Cells. Mol. Nutr. Food Res..

[45] Arumugam T., Ghazi T., Chuturgoon A.A. (2021). Fumonisin B1 alters global m6A RNA methylation and epigenetically regulates Keap1-Nrf2 signaling in human hepatoma (HepG2) cells. Arch. Toxicol..

[46] Huang T.T., Kudo N., Yoshida M., Miyamoto S. (2000). A nuclear export signal in the N-terminal regulatory domain of IκBα controls cytoplasmic localization of inactive NF-κB/IκBα complexes. Proc. Natl. Acad. Sci. USA.

[47] Solan N.J., Miyoshi H., Carmona E.M., Bren G.D., Paya C.V. (2002). RelB Cellular Regulation and Transcriptional Activity Are Regulated by p100. J. Biol. Chem..

[48] Jardin F. (2022). NFkB Pathway and Hodgkin Lymphoma. Biomedicines.

[49] KEGG PATHWAY: NF-Kappa B Signaling Pathway—Reference Pathway’. https://www.kegg.jp/pathway/map=map04064&keyword=nfkb.

[50] Savinova O.V., Hoffmann A., Ghosh G. (2009). The Nfkb1 and Nfkb2 Proteins p105 and p100 Function as the Core of High-Molecular-Weight Heterogeneous Complexes. Mol. Cell.

[51] Sun S.-C. (2017). The non-canonical NF-κB pathway in immunity and inflammation. Nat. Rev. Immunol..

[52] Kayagaki N., Yan M., Seshasayee D., Wang H., Lee W., French D.M., Grewal I.S., Cochran A.G., Gordon N.C., Yin J. (2002). BAFF/BLyS Receptor 3 Binds the B Cell Survival Factor BAFF Ligand through a Discrete Surface Loop and Promotes Processing of NF-κB2. Immunity.

[53] Fagiani F., Catanzaro M., Buoso E., Basagni F., Di Marino D., Raniolo S., Amadio M., Frost E.H., Corsini E., Racchi M. (2020). Targeting Cytokine Release Through the Differential Modulation of Nrf2 and NF-κB Pathways by Electrophilic/Non-Electrophilic Compounds. Front. Pharmacol..

[54] Zhang T., Ma C., Zhang Z., Zhang H., Hu H. (2021). NF-κB signaling in inflammation and cancer. Medcomm.

[55] Medeiros M., Candido M.F., Valera E.T., Brassesco M.S. (2021). The multifaceted NF-kB: Are there still prospects of its inhibition for clinical intervention in pediatric central nervous system tumors?. Cell. Mol. Life Sci..

[56] Bian X., McAllister-Lucas L.M., Shao F., Schumacher K.R., Feng Z., Porter A.G., Castle V.P., Opipari A.W. (2001). NF-κB Activation Mediates Doxorubicin-induced Cell Death in N-type Neuroblastoma Cells. J. Biol. Chem..

[57] Brandetti E., Focaccetti C., Pezzolo A., Ognibene M., Folgiero V., Cotugno N., Benvenuto M., Palma P., Manzari V., Rossi P. (2021). Enhancement of Neuroblastoma NK-Cell-Mediated Lysis through NF-kB p65 Subunit-Induced Expression of FAS and PVR, the Loss of Which Is Associated with Poor Patient Outcome. Cancers.

[58] Posadas I., Santos P., Ceña V. (2012). Acetaminophen Induces Human Neuroblastoma Cell Death through NFKB Activation. PLoS ONE.

[59] Karacay B., Sanlioglu S., Griffith T.S., Sandler A., Bonthius D.J. (2004). Inhibition of the NF-κB pathway enhances TRAIL-mediated apoptosis in neuroblastoma cells. Cancer Gene Ther..

[60] Lorenzi S., Forloni M., Cifaldi L., Antonucci C., Citti A., Boldrini R., Pezzullo M., Castellano A., Russo V., van der Bruggen P. (2012). IRF1 and NF-kB Restore MHC Class I-Restricted Tumor Antigen Processing and Presentation to Cytotoxic T Cells in Aggressive Neuroblastoma. PLoS ONE.

[61] Forloni M., Albini S., Limongi M.Z., Cifaldi L., Boldrini R., Nicotra M.R., Giannini G., Natali P.G., Giacomini P., Fruci D. (2010). NF-κB, and not MYCN, Regulates MHC Class I and Endoplasmic Reticulum Aminopeptidases in Human Neuroblastoma Cells. Cancer Res..

[62] Nakshatri H., Appaiah H.N., Anjanappa M., Gilley D., Tanaka H., Badve S., Crooks P.A., Mathews W., Sweeney C., Bhat-Nakshatri P. (2015). NF-κB-dependent and -independent epigenetic modulation using the novel anti-cancer agent DMAPT. Cell Death Dis..

[63] Pattyn A., Morin X., Cremer H., Goridis C., Brunet J.-F. (1999). The homeobox gene Phox2b is essential for the development of autonomic neural crest derivatives. Nature.

[64] Ooi C.Y., Carter D.R., Liu B., Mayoh C., Beckers A., Lalwani A., Nagy Z., De Brouwer S., Decaesteker B., Hung T.-T. (2018). Network Modeling of microRNA–mRNA Interactions in Neuroblastoma Tumorigenesis Identifies miR-204 as a Direct Inhibitor of MYCN. Cancer Res..

[65] Perri P., Ponzoni M., Corrias M.V., Ceccherini I., Candiani S., Bachetti T. (2021). A Focus on Regulatory Networks Linking MicroRNAs, Transcription Factors and Target Genes in Neuroblastoma. Cancers.

[66] Flora A., Lucchetti H., Benfante R., Goridis C., Clementi F., Fornasari D. (2001). SP Proteins and PHOX2B Regulate the Expression of the Human*PHOX2a*Gene. J. Neurosci..

[67] Di Zanni E., Bianchi G., Ravazzolo R., Raffaghello L., Ceccherini I., Bachetti T. (2017). Targeting of*PHOX2B*expression allows the identification of drugs effective in counteracting neuroblastoma cell growth. Oncotarget.

[68] Amiel J., Laudier B., Attié-Bitach T., Trang H., de Pontual L., Gener B., Trochet D., Etchevers H., Ray P., Simonneau M. (2003). Polyalanine expansion and frameshift mutations of the paired-like homeobox gene PHOX2B in congenital central hypoventilation syndrome. Nat. Genet..

[69] Mosse Y.P., Laudenslager M., Khazi D., Carlisle A.J., Winter C.L., Rappaport E., Maris J.M. (2004). Germline PHOX2B Mutation in Hereditary Neuroblastoma. Am. J. Hum. Genet..

[70] Zhao J., Zhu Y., Xie X., Yao Y., Zhang J., Zhang R., Huang L., Cheng J., Xia H., He J. (2019). Pleiotropic effect of common PHOX2B variants in Hirschsprung disease and neuroblastoma. Aging.

[71] Di Lascio S., Benfante R., Cardani S., Fornasari D. (2021). Research Advances on Therapeutic Approaches to Congenital Central Hypoventilation Syndrome (CCHS). Front. Neurosci..

[72] van Limpt V., Chan A., Schramm A., Eggert A., Versteeg R. (2005). Phox2B mutations and the Delta–Notch pathway in neuroblastoma. Cancer Lett..

[73] Hung Y.P., Lee J.P., Bellizzi A.M., Hornick J.L. (2017). PHOX2B reliably distinguishes neuroblastoma among small round blue cell tumours. Histopathology.

[74] Stutterheim J., Gerritsen A., Zappeij-Kannegieter L., Kleijn I., Dee R., Hooft L., van Noesel M.M., Bierings M., Berthold F., Versteeg R. (2008). *PHOX2B* Is a Novel and Specific Marker for Minimal Residual Disease Testing in Neuroblastoma. J. Clin. Oncol..

[75] Longo L., Borghini S., Schena F., Parodi S., Albino D., Bachetti T., Da Prato L., Truini M., Gambini C., Tonini G.P. (1992). PHOX2A and PHOX2B genes are highly co-expressed in human neuroblastoma. Int. J. Oncol..

[76] Trochet D., Bourdeaut F., Janoueix-Lerosey I., Deville A., de Pontual L., Schleiermacher G., Coze C., Philip N., Frébourg T., Munnich A. (2004). Germline Mutations of the Paired–Like Homeobox 2B (PHOX2B) Gene in Neuroblastoma. Am. J. Hum. Genet..

[77] Di Lascio S., Bachetti T., Saba E., Ceccherini I., Benfante R., Fornasari D. (2012). Transcriptional dysregulation and impairment of PHOX2B auto-regulatory mechanism induced by polyalanine expansion mutations associated with congenital central hypoventilation syndrome. Neurobiol. Dis..

[78] Di Lascio S., Benfante R., Di Zanni E., Cardani S., Adamo A., Fornasari D., Ceccherini I., Bachetti T. (2017). Structural and functional differences in*PHOX2B*frameshift mutations underlie isolated or syndromic congenital central hypoventilation syndrome. Hum. Mutat..

[79] Revet I., Huizenga G., Chan A., Koster J., Volckmann R., van Sluis P., Øra I., Versteeg R., Geerts D. (2008). The MSX1 homeobox transcription factor is a downstream target of PHOX2B and activates the Delta–Notch pathway in neuroblastoma. Exp. Cell Res..

[80] Chen Y., Takita J., Choi Y.L., Kato M., Ohira M., Sanada M., Wang L., Soda M., Kikuchi A., Igarashi T. (2008). Oncogenic mutations of ALK kinase in neuroblastoma. Nature.

[81] Pugh T.J., Morozova O., Attiyeh E.F., Asgharzadeh S., Wei J.S., Auclair D., Carter S.L., Cibulskis K., Hanna M., Kiezun A. (2013). The genetic landscape of high-risk neuroblastoma. Nat. Genet..

[82] Ke X.-X., Zhang D., Zhao H., Hu R., Dong Z., Yang R., Zhu S., Xia Q., Ding H.-F., Cui H. (2015). Phox2B correlates with MYCN and is a prognostic marker for neuroblastoma development. Oncol. Lett..

[83] Reiff T., Tsarovina K., Majdazari A., Schmidt M., del Pino I., Rohrer H. (2010). Neuroblastoma Phox2b Variants Stimulate Proliferation and Dedifferentiation of Immature Sympathetic Neurons. J. Neurosci..

[84] Naftali O., Maman S., Meshel T., Sagi-Assif O., Ginat R., Witz I.P. (2016). PHOX2B is a suppressor of neuroblastoma metastasis. Oncotarget.

[85] Maman S., Edry-Botzer L., Sagi-Assif O., Meshel T., Yuan W., Lu W., Witz I.P. (2013). The metastatic microenvironment: Lung-derived factors control the viability of neuroblastoma lung metastasis. Int. J. Cancer.

[86] de Pontual L., Trochet D., Bourdeaut F., Thomas S., Etchevers H., Chompret A., Minard V., Valteau D., Brugieres L., Munnich A. (2007). Methylation-associated PHOX2B gene silencing is a rare event in human neuroblastoma. Eur. J. Cancer.

[87] Iyer N.G., Özdag H., Caldas C. (2004). p300/CBP and cancer. Oncogene.

[88] Yerra V.G., Negi G., Sharma S.S., Kumar A. (2013). Potential therapeutic effects of the simultaneous targeting of the Nrf2 and NF-κB pathways in diabetic neuropathy. Redox Biol..

[89] Sedighi M., Baluchnejadmojarad T., Afshin-Majd S., Amiri M., Aminzade M., Roghani M. (2020). Anti-aging Klotho Protects SH-SY5Y Cells Against Amyloid β1–42 Neurotoxicity: Involvement of Wnt1/pCREB/Nrf2/HO-1 Signaling. J. Mol. Neurosci..

[90] Alvariño R., Alonso E., Tabudravu J.N., Pérez-Fuentes N., Alfonso A., Botana L.M. (2020). Tavarua Deoxyriboside A and Jasplakinolide as Potential Neuroprotective Agents: Effects on Cellular Models of Oxidative Stress and Neuroinflammation. ACS Chem. Neurosci..

[91] Jaafaru M.S., Nordin N., Rosli R., Shaari K., Bako H.Y., Saad N., Noor N.M., Razis A.F.A. (2019). Neuroprotective effects of glucomoringin-isothiocyanate against H2O2-Induced cytotoxicity in neuroblastoma (SH-SY5Y) cells. NeuroToxicology.

[92] Holla V.R., Elamin Y.Y., Bailey A.M., Johnson A.M., Litzenburger B.C., Khotskaya Y.B., Sanchez N.S., Zeng J., Shufean A., Shaw K.R. (2017). ALK: A tyrosine kinase target for cancer therapy. Mol. Case Stud..

[93] Nakagawara A., Li Y., Izumi H., Muramori K., Inada H., Nishi M. (2018). Neuroblastoma. Jpn. J. Clin. Oncol..

[94] Heukamp L.C., Thor T., Schramm A., De Preter K., Kumps C., De Wilde B., Odersky A., Peifer M., Lindner S., Spruessel A. (2012). Targeted Expression of Mutated ALK Induces Neuroblastoma in Transgenic Mice. Sci. Transl. Med..

[95] Lopez-Delisle L., Pierre-Eugène C., Louis-Brennetot C., Surdez D., Raynal V., Baulande S., Boeva V., Grossetête-Lalami S., Combaret V., Peuchmaur M. (2018). Activated ALK signals through the ERK–ETV5–RET pathway to drive neuroblastoma oncogenesis. Oncogene.

[96] Cage T.A., Chanthery Y., Chesler L., Grimmer M., Knight Z., Shokat K., Weiss W.A., Gustafson W.C. (2015). Downregulation of MYCN through PI3K Inhibition in Mouse Models of Pediatric Neural Cancer. Front. Oncol..

[97] Guo Y., Guo D., Zhang S., Zhang Y., He X., Jiang X., Chan A.M.-L., Zou L., Sun J., Zhao H. (2022). Inhibition of PI3 kinase isoform p110α suppresses neuroblastoma growth and induces the reduction of Anaplastic Lymphoma Kinase. Cell Biosci..

[98] Opel D., Poremba C., Simon T., Debatin K.-M., Fulda S. (2007). Activation of Akt Predicts Poor Outcome in Neuroblastoma. Cancer Res..

[99] Heiss A., Ammer H., Eisinger D.A. (2009). δ-Opioid receptor-stimulated Akt signaling in neuroblastoma×glioma (NG108-15) hybrid cells involves receptor tyrosine kinase-mediated PI3K activation. Exp. Cell Res..

[100] Jin L., Mi T., Wu X., Wang Z., Zhang Z., Liu J., Wang Z., Wang J., Li M., Ren C. (2023). BI-D1870 Induces Mitotic Dysfunction and Apoptosis in Neuroblastoma by Regulating the PI3K-Akt-mTORC1 Signal Axis. Cancers.

[101] Fulda S. (2008). Tumor resistance to apoptosis. Int. J. Cancer.

[102] Di Zanni E., Fornasari D., Ravazzolo R., Ceccherini I., Bachetti T. (2015). Identification of novel pathways and molecules able to down-regulate PHOX2B gene expression by in vitro drug screening approaches in neuroblastoma cells. Exp. Cell Res..

[103] Tetri L.H., Kolla V., Golden R.L., Iyer R., Croucher J.L., Choi J., Macfarland S.P., Naraparaju K., Guan P., Nguyen F. (2020). RET receptor expression and interaction with TRK receptors in neuroblastomas. Oncol. Rep..

[104] Müller C.M., Haase M.G., Kemnitz I., Fitze G. (2014). Genetic mosaicism of a frameshift mutation in the RET gene in a family with Hirschsprung disease. Gene.

[105] Lambertz I., Kumps C., Claeys S., Lindner S., Beckers A., Janssens E., Carter D.R., Cazes A., Cheung B.B., De Mariano M. (2015). Upregulation of MAPK Negative Feedback Regulators and RET in Mutant ALK Neuroblastoma: Implications for Targeted Treatment. Clin. Cancer Res..

[106] Zhang C., Su Z.-Y., Khor T.O., Shu L., Kong A.-N.T. (2013). Sulforaphane enhances Nrf2 expression in prostate cancer TRAMP C1 cells through epigenetic regulation. Biochem. Pharmacol..

[107] Szklarczyk D., Kirsch R., Koutrouli M., Nastou K., Mehryary F., Hachilif R., Gable A.L., Fang T., Doncheva N.T., Pyysalo S. (2022). The STRING database in 2023: Protein–protein association networks and functional enrichment analyses for any sequenced genome of interest. Nucleic Acids Res..

[108] Lombardo S.D., Presti M., Mangano K., Petralia M.C., Basile M.S., Libra M., Candido S., Fagone P., Mazzon E., Nicoletti F. (2019). Prediction of PD-L1 Expression in Neuroblastoma via Computational Modeling. Brain Sci..

